# Metabolomics approach in the investigation of depression biomarkers in pharmacologically induced immune-related depression

**DOI:** 10.1371/journal.pone.0208238

**Published:** 2018-11-29

**Authors:** Andreas Baranyi, Andreas Meinitzer, Hans-Bernd Rothenhäusler, Omid Amouzadeh-Ghadikolai, Dirk V. Lewinski, Robert J. Breitenecker, Markus Herrmann

**Affiliations:** 1 Department of Psychiatry and Psychotherapeutic Medicine, Medical University of Graz, Graz, Austria; 2 Clinical Institute of Medical and Chemical Laboratory Diagnostics, Medical University of Graz, Graz, Austria; 3 LKH Graz Süd-West, Graz, Austria; 4 Division of Cardiology, Department of Internal Medicine, Medical University of Graz, Graz, Austria; 5 Institute of Innovation Management, Johannes Kepler University, Linz, Austria; University of California San Francisco, UNITED STATES

## Abstract

**Background:**

The aim of this study was to identify previously unrecognised biological pathways and biomarkers that might expand the inflammatory hypothesis of depression.

**Methods:**

Broad metabolomics analyses in plasma samples from 31 chronic hepatitis C-infected patients with and without immune-related depression were carried out using the Absolute IDQ p180 kit—a targeted metabolomics approach of combined direct flow injection and liquid chromatography that measures acylcarnitines, amino acids, biogenic amines, glycerophospholipids, sphingolipids, and sugars.

**Results:**

The measurements showed that the average concentration of the branched-chain amino acid isoleucine was significantly lower in depressive HCV patients in comparison to non-depressive HCV patients [depression group: Median 51.35 (43.4–60.2 μmol/L) vs. Median 62.10 (38.4–81.7 μmol/L); U = -2.958; p = 0.002]. All other amino acids, acylcarnitines, biogenic amines, glycerophospholipids, sphingolipids, sugars, liver enzymes and thyroid levels showed no statistically significant differences.

**Conclusions:**

The results of the present study suggest that the branched-chain amino acid isoleucine might play a role in the pathophysiology of immune-related major depression, which expands existing knowledge about inflammatory hypothesis of depression.

## Introduction

According to the WHO (2017), worldwide more than 300 million people of all ages suffer from depression, and globally, depression is the leading cause of disability. Thus, depressive illness is one of the major contributors to the overall global disease burden and is one of the priority conditions covered by the WHO’s Mental Health Gap Action Programme (mhGAP) [1]. Depressive patients often suffer from pronounced emotional, somatic, and functional impairments [2,3].

Several pathomechanistic theories for major depression have been proposed. According to the classic monoaminergic hypothesis, depression is the result of a dysfunction of the noradrenaline and 5-hydroxytryptamine (5-HT) mediated neurotransmission. Another well-known concept of major depression is the hyperactivity of the hypothalamic-pituitary-adrenal (HPA) axis. However, both these concepts are not sufficient to explain comprehensively the pathophysiological processes leading to mood imbalances and major depression. Newer models focus on the alterations of neuroplasticity and neurotrophins or the involvement of inflammatory processes [4]. The inflammatory hypothesis of depression proposes that inflammatory cytokines, such as interferon (IFN)-α, IFN-γ, and tumor necrosis factor-α (TNF-α), increase the activity of the enzyme indoleamine 2,3-dioxygenase (IDO), leading to an activation of the catabolism of the serotonin precursor tryptophan to kynurenine [5–9]. The resulting larger amount of kynurenine crosses the blood-brain barrier and is broken down into its neurotoxic metabolites, 3-hydroxykynurenine (3-HK) and quinolinic acid. Quinolinic acid has strong agonistic effects on the N-methyl-D-aspartate (NMDA) receptor. The NMDA receptor is a glutamate receptor that can induce neuronal damage upon overstimulation [6–9]. Considering the pathophysiological heterogeneity of major depression, the most effective therapy might differ between various forms of the disease. The correct identification of underlying biochemical causes might be a prerequisite for efficient treatment strategies in different forms of major depression [10, 11]. Here we aimed to identify new biomarkers for immune-related depression using the model of pharmacologically induced immune-related depression (PIRD). PIRD is a well-established condition that is often used to study the inflammatory hypothesis of depression in humans.

A prototype of PIRD is the IFN-α induced depression in hepatitis C (HCV) patients. Even in the era of directly acting antivirals (DAAs) [12], IFN-α induced depression still remains an important model to study inflammatory pathways in depression and other mental disease. In the present study we aimed to identify new biomarkers for immune-related depression in IFN-α treated depressive HCV patients using targeted metabolomics. This approach allows the simultaneous analysis of multiple small molecular compounds including acylcarnitines, amino acids, biogenic amines, glycerophospholipids, sphingolipids, and sugars. The results provide a comprehensive overview of an individual’s metabolic status and may help to explore new biochemical markers for PIRD.

## Methods

### Study design

We investigated 31 hepatitis C patients treated with IFN-α who had participated in a previous research project on immune-related depression [5, 6, 13, 14]. Detailed information about the primary study has been published before [5]. All patients were treated in the outpatient clinic of the Division of Gastroenterology and Hepatology, Department of Internal Medicine, Medical University of Graz, Austria. At baseline and after one month of IFN-α therapy, patients were assessed in a clinical psychiatric interview performed by an experienced consultation-liaison psychiatrist (A.B.) for the presence of depressive symptoms. In addition, at baseline and after one month of IFN-α therapy all patients were evaluated with the psychometric observer-rated scale Hamilton Depression Rating Scale (HAMD-17, Hamilton, 1967 [15]). One month after the initial IFN-α therapy the diagnosis of major depression required the diagnosis of major depression in a clinical psychiatric interview performed by an experienced consultation-liaison psychiatrist (A.B.) and additionally the exceeding of the cut-offs of the HAMD-17 [15] and the self- reporting Beck Depression Inventory (BDI, Beck et al., 1961 [16]). After the making of the clinical diagnosis in the clinical psychiatric interview the consultation-liaison psychiatrist (A.B.) had also access to the scores of the HAMD-17 and BDI.

Fasting plasma samples were collected between 9 and 10am one month after the commencement of IFN-α therapy. All samples were centrifuged within 0.5 hours after blood collection and stored until batched analysis. These samples were used for metabolomics analysis. The study was approved by the Institutional Review Board of the University of Medicine of Graz, Austria, and all patients gave their written informed consent. All methods were performed in accordance with the relevant guidelines and regulations.

### Participants

Participants were recruited through the outpatient clinic of the Division of Gastroenterology and Hepatology, Department of Internal Medicine, Medical University of Graz, Austria. A detailed description of the study cohort has been published before [5, 6, 13]. All patients had chronic hepatitis C infection and were treated with standard pegylated (peg) IFN-α therapy. Exclusion criteria from enrolment were as follows: (1) chronic liver diseases other than HCV, (2) other somatic co-morbidities (i.e. diabetes, cardiovascular disease, cancer), and/or (3) a former diagnosis of any neurological disease.

### Metabolomics analyses

Plasma samples were analysed with the Absolute IDQ p180 kit (BIOCRATES Life Sciences AG, Innsbruck, Austria) [17] on a SCIEX4000 triple quadrupole mass spectrometer (MS/MS; SCIEX) coupled to an Agilent 1100 liquid chromatography (LC) system equipped with a Zorbax Eclipse C18 150x4.6 mm, 5 μm column (Agilent, Palo Alto, USA).

The Absolute IDQ p180 kit measures 186 analytes: acylcarnitines, amino acids, biogenic amines, glycerophospholipids, sphingolipids and sugars, using a combined approach of liquid chromatography tandem mass spectrometry (LC-MS/MS) and direct flow injection mass spectrometry (DFI-MS/MS). DFI-MS/MS bypasses the LC system by injecting samples directly into the MS/MS [17].

Prior to analysis samples were prepared as follows: 10 μl of the internal standard solution supplied with the kit and 10 μl of plasma were transferred to each well of a 96-well filterspot extraction plate. After drying the plate under a gentle stream of nitrogen, samples were derivatized with phenyl-isothiocyanate (PITC). Subsequently the plate was dried again and samples were eluted with 5 mM ammonium acetate in methanol. 100 μl of the extracts were diluted with 200 μl of 40% methanol in water for LC-MS/MS analysis. In addition, 10μl of extract were mixed with 190μl of kit running solvent (Biocrates Life Sciences AG) for DFI-MS/MS.

Analytes with results under the limit of detection (LOD) and analytes with insufficient validation for human plasma have been excluded. Analytes with levels between the lower limit of quantification (LOQ) and the limit of detection (LOD) are marked in Table 1 by a bullet. In accordance with the BIOCRATES guidelines of the Absolute IDQ p180 kit (BIOCRATES Life Sciences, 2010) [17], analytes with a semi-quantitative determination are mentioned in a separate column of Table 1.

**Table 1:** Depression questionnaires, thyroid levels, liver enzymes and metabolomic analyses (Absolute IDQ p180kit) one month after the initial IFN-α treatment.

	Patients without Depression (n = 21)				Patients with Depression (n = 10)				Diff. of Median values ^4^	Semi-quanti-tative	Mann-Whitney-U Test			Benjamini Hochberg Critical Value ^3^
	Median	Range ^5^	Mean	SD	Median	Range ^5^	Mean	SD			Mann-Whitney-U	U	p (exact)	
**Depression Questionnaires**														
BDI ^1^	5.0	8.0	3.91	2.28	17.0	31.0	19.2	9.11	12.00		0	-4.468	0.00**	**0.002**
HAMD-17 ^2^	4.0	10.0	4.14	3.15	14.5	11.0	15.0	3.62	10.50		0	-4.449	0.00**	**0.004**
**Thyroid Levels**														
Thyroid-stimulating hormone [mU/L]	1.54	3.01	1.60	0.75	1.12	2.21	1.19	0.75	-0.42		42	-1.162	0.265	0.049
Free thyroxine [pmol/L]	13.5	7.4	13.19	2.19	12.2	5.5	13.54	2.31	-1.30		59.5	-0.032	0.975	0.234
Free triiodothyronine [pmol/L]	4.4	2.8	4.61	0.73	4.7	5.7	4.99	1.76	0.30		55.5	-0.291	0.776	0.194
**Liver Enzymes**														
Bilirubin [mg/dL]	0.71	2.81	0.82	0.61	0.79	2.9	1.56	1.21	0.08		53	-1.879	0.063	0.011
Alkaline phosphatase [U/L]	66.0	70.0	68.80	15.72	69.0	45.0	66.0	14.89	3.00		87	-0.142	0.908	0.219
Gamma-glutamyltransferase [U/L]	32.0	115.0	43.67	31.02	37.0	156.0	57.89	48.71	5.00		70.5	-1.087	0.283	0.054
Aspartate transaminase [U/L]	39.0	67.0	42.52	16.95	38.0	57.0	38.56	17.13	-1.00		77.5	-0.771	0.449	0.103
Alanine transaminase [U/L]	38.0	92.0	45.05	25.2	28.0	68.0	40.67	24.34	-10.00		80	-0.657	0.533	0.124
Cholinesterase [U/L]	8422.0	8531.0	8103.0	2351.4	10276.5	4433.0	9518.0	2085.7	1854.5		12	-1.131	0.304	0.061
**Metabolomic Analyses—Absolute IDQ p180kit** **Acylcarnitines**														
C0 (Carnitine) [μmol/L]	37.01	33.63	37.58	9.18	34.11	14.52	33.29	4.05	-2.90		84	-0.887	0.393	0.094
C2 (Acetylcarnitine) [μmol/L]	5.11	8.22	5.55	2.10	5.37	4.98	5.42	1.59	0.26		102	-0.127	0.917	0.221
C3 (Propionylcarnitine) [μmol/L] ^●^	0.23	0.26	0.22	0.05	0.17	0.1	0.18	0.03	-0.06					
C4 (Butyrylcarnitine) [μmol/L] ^●^	0.15	0.16	0.15	0.04	0.12	0.19	0.14	0.06	-0.03					
C5 (Valerylcarnitine) [μmol/L] ^●^	0.13	0.36	0.19	0.11	0.1	0.35	0.17	0.12	-0.03					
C9 (Nonaylcarnitine) [μmol/L]	0.05	0.06	0.05	0.01	0.04	0.04	0.05	0.01	-0.01	X	94	-0.465	0.663	0.156
C14-1 (Tetradecenoylcarnitine) [μmol/L]	0.13	0.17	0.13	0.04	0.16	0.13	0.15	0.05	0.03	X	74	-1.31	0.201	0.031
C16 (Hexadecanoylcarnitine) [μmol/L] ^●^	0.10	0.1	0.11	0.03	0.13	0.14	0.13	0.05	0.03					
C18 (Octadecanoylcarnitine) [μmol/L] ^●^	0.04	0.08	0.05	0.02	0.04	0.05	0.05	0.02	0.00					
C18:1 (Octadecenoylcarnitine) [μmol/L]	0.13	0.18	0.14	0.05	0.15	0.22	0.16	0.07	0.02	X	89	-0.676	0.519	0.119
C18:2 (Octadecadienylcarnitine) [μmol/L]	0.06	0.08	0.06	0.02	0.06	0.1	0.06	0.03	0.00	X	98	-0.296	0.787	0.196
**Amino Acids**														
Alanine [μmol/L]	290.0	293.0	312.24	83.0	288.0	164.0	293.6	51.14	-2.00		94.5	-0.444	0.663	0.158
Arginine [μmol/L]	81.0	56.4	84.04	16.0	76.7	63.8	74.84	22.07	-4.30		76.5	-1.205	0.233	0.038
Asparagine [μmol/L]	39.1	33.8	39.09	8.03	32.8	25.0	34.76	7.83	-6.30		77	-1.183	0.25	0.045
Glutamine [μmol/L]	463.0	450.0	442.29	120.59	437.5	330.0	428.3	100.38	-25.50		101	-0.169	0.884	0.212
Glutamate [μmol/L]	283.0	439.0	330.52	131.13	283.0	354.0	332.1	112.36	0.00		101	-0.169	0.884	0.214
Glycine [μmol/L]	207.0	167.0	214.33	44.14	189.5	141.0	208.3	48.61	-17.50		88.5	-0.697	0.492	0.110
Isoleucine [μmol/L]	62.1	43.3	62.0	11.30	51.35	16.8	51.07	5.48	-10.75		35	-2.958	0.002**	**0.005**
Leucine [μmol/L]	111.0	101.2	116.31	21.85	99.8	43.9	98.48	14.85	-11.20		51	-2.283	0.022	0.007
Lysine [μmol/L]	170.0	94.0	171.76	21.91	161.0	115.0	167.4	35.92	-9.00		88	-0.719	0.492	0.112
Methionine [μmol/L]	18.9	32.17	19.41	6.37	16.45	13.4	17.32	4.66	-2.45		82	-0.972	0.348	0.074
Ornithine [μmol/L]	61.8	85.6	65.09	19.66	60.5	54.8	63.79	17.75	-1.30		97	-0.338	0.755	0.185
Phenylalanine [μmol/L]	70.20	75.7	73.75	18.24	67.65	27.0	65.81	8.23	-2.55		81	-1.014	0.327	0.068
Proline [μmol/L]	210.0	271.0	214.1	69.42	174.5	151.0	175.8	41.60	-35.50		69	-1.521	0.135	0.022
Serine [μmol/L]	106.0	60.4	104.06	16.21	109.0	64.2	109.81	21.97	3.00		90	-0.634	0.546	0.126
Threonine [μmol/L]	125.0	106.6	134.77	31.13	135.5	153.2	132.94	45.17	10.50		94.5	-0.444	0.663	0.160
Tryptophan [μmol/L]	55.2	29.7	52.9	8.84	51.2	21.2	50.84	6.02	-4.00		80	-1.057	0.306	0.063
Tyrosine [μmol/L]	76.2	111.8	77.63	25.03	67.95	39.8	75.01	15.33	-8.25		98	-0.296	0.787	0.198
Valine [μmol/L]	227.0	129.0	232.29	37.46	199.0	69.0	202.8	25.03	-28.00		55.5	-2.092	0.035	0.009
**Biogenic Amines**														
Creatinine [μmol/L]	75.6	40.10	74.74	12.52	71.8	49.9	71.15	15.93	-3.80		91.5	0.571	0.574	0.138
Putrescine [μmol/L] ^●^	0.13	0.13	0.14	0.04	0.16	0.2	0.18	0.07	0.03					
Sarcosine [μmol/L]	7.92	6.41	8.03	1.69	6.8	6.87	7.85	2.60	-1.12		84	-0.887	0.393	0.095
Taurine [μmol/L]	46.4	49.40	52.83	14.92	44.4	38.5	47.76	12.60	-2.00		81	1.014	0.327	0.070
**Glycerophospholipids**														
lysoPhosphatidylcholine acyl C14:0 [μmol/L]	2.67	2.69	2.98	0.72	2.87	1.23	3.10	0.51	0.20	X	79	-1.099	0.287	0.056
lysoPhosphatidylcholine acyl C16:0 [μmol/L]	97.86	98.54	102.3	29.09	92.62	110.44	109.37	38.86	-5.24	X	104	-0.042	0.983	0.236
lysoPhosphatidylcholine acyl C16:1 [μmol/L]	2.15	1.91	2.27	0.59	2.4	1.76	2.5	0.57	0.25	X	75	-1.268	0.217	0.034
lysoPhosphatidylcholine acyl C17:0 [μmol/L]	1.95	3.12	2.14	0.83	2.15	2.89	2.38	0.94	0.20	X	92	-0.549	0.603	0.146
lysoPhosphatidylcholine acyl C18:0 [μmol/L]	39.84	46.59	40.45	13.14	33.32	40.6	41.89	15.13	-6.52	X	103	-0.085	0.95	0.228
lysoPhosphatidylcholine acyl C18:1 [μmol/L]	21.99	25.31	21.79	6.96	19.14	19.7	22.4	7.94	-2.85	X	104	-0.042	0.983	0.237
lysoPhosphatidylcholine acyl C18:2 [μmol/L]	27.81	34.41	29.99	10.23	25.75	35.51	29.45	12.73	-2.06	X	94	-0.465	0.663	0.162
lysoPhosphatidylcholine acyl C20:3 [μmol/L]	2.65	2.36	2.52	0.76	1.87	2.05	2.21	0.75	-0.78	X	80	-1.056	0.306	0.065
lysoPhosphatidylcholine acyl C20:4 [μmol/L]	5.32	10.15	6.76	2.81	4.96	8.62	6.47	2.86	-0.36	X	90	-0.634	0.546	0.128
lysoPhosphatidylcholine acyl C24:0 [μmol/L]	0.44	0.64	0.45	0.16	0.48	0.29	0.45	0.11	0.04	X	103	-0.085	0.95	0.230
lysoPhosphatidylcholine acyl C26:0 [μmol/L]	0.91	1.38	0.94	0.36	0.8	0.84	0.89	0.27	-0.11	X	99	-0.254	0.819	0.205
lysoPhosphatidylcholine acyl C26:1 [μmol/L]	0.29	0.36	0.29	0.1	0.25	0.23	0.26	0.09	-0.04	X	95	-0.423	0.693	0.167
lysoPhosphatidylcholine acyl C28:0 [μmol/L]	1.09	1.32	1.08	0.36	0.88	0.74	0.97	0.26	-0.21	X	82	-0.972	0.348	0.076
lysoPhosphatidylcholine acyl C28:1 [μmol/L]	1.0	1.14	0.94	0.32	0.88	0.73	0.92	0.22	-0.12	X	103	-0.085	0.95	0.232
Phosphatidylcholine diacyl C24:0 [μmol/L]	0.28	0.38	0.26	0.1	0.23	0.24	0.25	0.08	-0.05	X	97	-0.338	0.755	0.187
Phosphatidylcholine diacyl C26:0 [μmol/L]	0.72	0.45	0.70	0.13	0.69	0.31	0.69	0.1	-0.03	X	96	-0.38	0.724	0.176
Phosphatidylcholine diacyl C28:1 [μmol/L]	2.17	2.55	2.37	0.71	2.63	1.72	2.52	0.46	0.46	X	81	-1.014	0.327	0.072
Phosphatidylcholine diacyl C30:0 [μmol/L]	3.09	5.22	3.45	1.42	3.4	2.71	3.79	0.91	0.31	X	77	-1.183	0.25	0.047
Phosphatidylcholine diacyl C32:0 [μmol/L]	20.12	14.45	19.67	3.83	21.43	5.75	21.35	1.52	1.31	X	64	-1.733	0.087	0.014
Phosphatidylcholine diacyl C32:1 [μmol/L]	15.84	39.54	19.15	8.83	18.19	30.06	23.27	10.79	2.35	X	78	-1.141	0.268	0.050
Phosphatidylcholine diacyl C32:2 [μmol/L]	3.2	7.24	3.51	1.67	3.8	2.96	3.65	0.93	0.60	X	83	-0.93	0.37	0.081
Phosphatidylcholine diacyl C32:3 [μmol/L]	0.5	0.73	0.55	0.17	0.56	0.33	0.57	0.09	0.06	X	82	-0.972	0.348	0.077
Phosphatidylcholine diacyl C34:1 [μmol/L]	181.6	195.50	192.03	41.89	198.44	83.15	194.73	21.46	16.84	X	88	-0.718	0.492	0.113
Phosphatidylcholine diacyl C34:2 [μmol/L]	355.18	286.48	365.98	70.26	360.62	113.16	367.49	38.36	5.44	X	104	-0.042	0.983	0.239
Phosphatidylcholine diacyl C34:3 [μmol/L]	19.08	21.0	20.06	5.88	20.24	9.35	20.37	2.64	1.16	X	82	-0.972	0.348	0.079
Phosphatidylcholine diacyl C34:4 [μmol/L]	1.22	1.72	1.37	0.48	1.27	0.81	1.32	0.25	0.05	X	97	-0.338	0.755	0.189
Phosphatidylcholine diacyl C36:0 [μmol/L]	3.64	4.68	3.92	1.2	3.95	3.23	3.98	0.99	0.31	X	97	-0.338	0.755	0.191
Phosphatidylcholine diacyl C36:1 [μmol/L]	31.88	26.01	31.51	7.61	32.28	11.38	31.39	3.65	0.40	X	102	-0.127	0.917	0.223
Phosphatidylcholine diacyl C36:2 [μmol/L]	198.21	151.56	206.56	43.47	207.11	78.68	207.07	27.59	8.90	X	104	-0.042	0.983	0.241
Phosphatidylcholine diacyl C36:3 [μmol/L]	112.24	153.13	121.2	34.07	110.55	52.38	111.03	15.11	-1.69	X	89	-0.676	0.519	0.121
Phosphatidylcholine diacyl C36:4 [μmol/L]	132.58	127.76	139.69	34.46	128.23	76.59	129.80	20.36	-4.35	X	96	-0.38	0.724	0.178
Phosphatidylcholine diacyl C36:5 [μmol/L]	13.9	13.41	15.48	4.19	14.63	7.64	15.0	2.20	0.73	X	99	-0.254	0.819	0.207
Phosphatidylcholine diacyl C36:6 [μmol/L]	1.07	1.48	1.12	0.32	1.07	0.46	1.08	0.14	0.00	X	104	-0.042	0.983	0.243
Phosphatidylcholine diacyl C38:0 [μmol/L]	3.36	3.33	3.5	0.78	3.49	2.21	3.76	0.82	0.13	X	85	-0.845	0.416	0.099
Phosphatidylcholine diacyl C38:1 [μmol/L]	2.83	4.81	2.80	1.16	2.7	2.72	2.75	0.86	-0.13	X	104	-0.042	0.983	0.245
Phosphatidylcholine diacyl C38:3 [μmol/L]	33.88	36.64	37.41	10.35	33.22	17.02	34.26	5.42	-0.66	X	96	-0.38	0.724	0.180
Phosphatidylcholine diacyl C38:4 [μmol/L]	67.46	74.59	69.81	18.52	63.86	34.29	65.36	10.08	-3.60	X	98	-0.296	0.787	0.200
Phosphatidylcholine diacyl C38:5 [μmol/L]	27.73	27.18	29.22	6.93	27.99	5.56	28.04	1.74	0.26	X	98	-0.296	0.787	0.201
Phosphatidylcholine diacyl C38:6 [μmol/L]	35.71	32.84	35.4	9.18	38.18	31.49	38.9	8.88	2.47	X	90	-0.634	0.546	0.129
Phosphatidylcholine diacyl C40:1 [μmol/L]	0.92	1.04	0.91	0.25	0.77	0.61	0.84	0.2	-0.15	X	83	-0.93	0.37	0.083
Phosphatidylcholine diacyl C40:2 [μmol/L]	1.7	1.6	1.57	0.41	1.41	1.28	1.48	0.36	-0.29	X	87	-0.761	0.466	0.104
Phosphatidylcholine diacyl C40:3 [μmol/L]	1.69	1.47	1.59	0.41	1.43	1.13	1.5	0.33	-0.26	X	96	-0.38	0.724	0.182
Phosphatidylcholine diacyl C40:4 [μmol/L]	3.32	2.22	3.29	0.66	3.08	1.15	3.14	0.31	-0.24	X	91	-0.592	0.574	0.140
Phosphatidylcholine diacyl C40:5 [μmol/L]	6.5	4.53	6.4	1.39	6.37	2.71	6.33	0.84	-0.13	X	95	-0.423	0.693	0.169
Phosphatidylcholine diacyl C40:6 [μmol/L]	13.79	12.95	14.2	3.94	14.64	9.31	15.65	3.08	0.85	X	83	-0.93	0.37	0.085
Phosphatidylcholine diacyl C42:0 [μmol/L]	0.83	0.62	0.81	0.17	0.73	0.52	0.76	0.18	-0.10	X	84	-0.887	0.393	0.097
Phosphatidylcholine diacyl C42:1 [μmol/L]	0.52	0.49	0.51	0.13	0.45	0.36	0.47	0.12	-0.07	X	87	-0.761	0.466	0.106
Phosphatidylcholine diacyl C42:2 [μmol/L]	0.58	0.55	0.58	0.15	0.58	0.34	0.56	0.12	0.00	X	95	-0.423	0.693	0.171
Phosphatidylcholine diacyl C42:4 [μmol/L]	0.59	0.64	0.61	0.15	0.63	0.16	0.6	0.07	0.04	X	92	-0.549	0.603	0.147
Phosphatidylcholine diacyl C42:5 [μmol/L]	0.58	0.57	0.6	0.14	0.64	0.19	0.62	0.07	0.06	X	73	-1.352	0.186	0.027
Phosphatidylcholine diacyl C42:6 [μmol/L]	0.57	0.66	0.57	0.13	0.6	0.16	0.57	0.06	0.03	X	93	-0.507	0.633	0.151
Phosphatidylcholine acyl-alkyl C30:0 [μmol/L]	0.48	0.45	0.49	0.13	0.54	0.27	0.52	0.09	0.06	X	91	-0.592	0.574	0.142
Phosphatidylcholine acyl-alkyl C30:2 [μmol/L]	0.26	0.37	0.25	0.09	0.23	0.26	0.24	0.08	-0.03	X	92	-0.549	0.603	0.149
Phosphatidylcholine acyl-alkyl C32:1 [μmol/L]	3.32	2.69	3.27	0.75	3.44	2.09	3.46	0.59	0.12	X	89	-0.676	0.519	0.122
Phosphatidylcholine acyl-alkyl C32:2 [μmol/L]	0.85	0.7	0.86	0.19	0.85	0.69	0.89	0.2	0.00	X	102	-0.127	0.917	0.225
Phosphatidylcholine acyl-alkyl C34:0 [μmol/L]	1.51	1.81	1.67	0.45	1.92	0.66	1.85	0.24	0.41	X	64	-1.733	0.087	0.016
Phosphatidylcholine acyl-alkyl C34:1 [μmol/L]	11.07	8.73	11.52	2.19	12.75	4.44	12.16	1.35	1.68	X	79	-1.099	0.287	0.058
Phosphatidylcholine acyl-alkyl C34:2 [μmol/L]	10.96	14.71	12.39	4.06	11.74	7.74	11.05	2.47	0.78	X	90	-0.634	0.546	0.131
Phosphatidylcholine acyl-alkyl C34:3 [μmol/L]	6.99	10.0	7.79	2.51	7.78	5.04	7.07	1.89	0.79	X	90	-0.634	0.546	0.133
Phosphatidylcholine acyl-alkyl C36:0 [μmol/L]	1.13	0.95	1.07	0.25	1.08	0.38	1.09	0.15	-0.05	X	95	-0.423	0.693	0.173
Phosphatidylcholine acyl-alkyl C36:1 [μmol/L]	13.07	14.37	12.99	3.63	12.97	6.29	12.85	2.21	-0.10	X	100	-0.211	0.852	0.210
Phosphatidylcholine acyl-alkyl C36:2 [μmol/L]	13.32	16.72	14.26	4.46	15.00	9.11	14.54	3.02	1.68	X	83	-0.93	0.37	0.086
Phosphatidylcholine acyl-alkyl C36:3 [μmol/L]	6.06	7.34	6.54	2.07	6.01	3.12	5.61	1.12	-0.05	X	87	-0.761	0.466	0.108
Phosphatidylcholine acyl-alkyl C36:4 [μmol/L]	16.84	24.66	18.67	5.82	16.67	15.00	16.77	5.02	-0.17	X	85	-0.845	0.416	0.101
Phosphatidylcholine acyl-alkyl C36:5 [μmol/L]	8.50	12.5	9.06	2.59	8.56	7.98	8.61	3.02	0.06	X	104	-0.042	0.983	0.246
Phosphatidylcholine acyl-alkyl C38:0 [μmol/L]	2.79	3.25	2.94	0.76	2.93	1.59	3.18	0.59	0.14	X	80	-1.056	0.306	0.067
Phosphatidylcholine acyl-alkyl C38:1 [μmol/L]	4.18	6.37	4.36	1.54	4.42	3.27	4.32	1.02	0.24	X	97	-0.338	0.755	0.192
Phosphatidylcholine acyl-alkyl C38:2 [μmol/L]	7.24	9.6	7.47	2.46	7.04	4.49	7.01	1.4	-0.20	X	99	-0.254	0.819	0.209
Phosphatidylcholine acyl-alkyl C38:3 [μmol/L]	9.22	8.41	9.51	2.43	8.91	4.01	8.72	1.19	-0.31	X	83	-0.93	0.37	0.088
Phosphatidylcholine acyl-alkyl C38:4 [μmol/L]	11.69	9.72	11.89	2.6	11.49	7.21	11.27	2.26	-0.20	X	94	-0.465	0.663	0.164
Phosphatidylcholine acyl-alkyl C38:5 [μmol/L]	13.09	14.34	13.19	3.52	12.63	9.10	12.55	3.29	-0.46	X	88	-0.718	0.492	0.115
Phosphatidylcholine acyl-alkyl C38:6 [μmol/L]	4.47	5.2	4.56	1.04	4.59	3.76	4.62	1.19	0.12	X	104	-0.042	0.983	0.248
Phosphatidylcholine acyl-alkyl C40:1 [μmol/L]	2.31	1.71	2.14	0.5	2.03	1.38	1.99	0.44	-0.28	X	78	-1.141	0.268	0.052
Phosphatidylcholine acyl-alkyl C40:2 [μmol/L]	2.78	2.44	2.88	0.67	2.89	1.88	2.87	0.58	0.11	X	102	-0.127	0.917	0.227
Phosphatidylcholine acyl-alkyl C40:3 [μmol/L]	4.26	4.50	4.39	1.06	4.15	2.83	4.12	0.75	-0.11	X	95	-0.423	0.693	0.174
Phosphatidylcholine acyl-alkyl C40:4 [μmol/L]	4.46	3.65	4.61	0.97	4.46	2.18	4.31	0.65	0.00	X	90	-0.634	0.546	0.135
Phosphatidylcholine acyl-alkyl C40:5 [μmol/L]	4.49	3.71	4.7	0.9	4.7	2.57	4.69	0.71	0.21	X	93	-0.507	0.633	0.153
Phosphatidylcholine acyl-alkyl C40:6 [μmol/L]	3.27	2.29	3.35	0.58	3.32	1.93	3.51	0.60	0.05	X	96	-0.38	0.724	0.183
Phosphatidylcholine acyl-alkyl C42:1 [μmol/L]	1.22	0.93	1.11	0.27	0.98	0.96	1.03	0.29	-0.24	X	83	-0.93	0.37	0.090
Phosphatidylcholine acyl-alkyl C42:2 [μmol/L]	1.12	0.96	1.04	0.27	0.97	0.78	0.98	0.24	-0.15	X	90	-0.634	0.546	0.137
Phosphatidylcholine acyl-alkyl C42:3 [μmol/L]	1.25	1.16	1.24	0.3	1.24	0.69	1.2	0.23	-0.01	X	91	-0.592	0.574	0.144
Phosphatidylcholine acyl-alkyl C42:4 [μmol/L]	1.43	1.17	1.4	0.29	1.29	0.85	1.26	0.26	-0.14	X	76	-1.225	0.233	0.040
Phosphatidylcholine acyl-alkyl C42:5 [μmol/L]	2.89	1.49	2.87	0.41	2.74	1.5	2.77	0.44	-0.15	X	88	-0.718	0.492	0.117
Phosphatidylcholine acyl-alkyl C44:3 [μmol/L]	0.37	0.38	0.37	0.1	0.39	0.25	0.37	0.09	0.02	X	101	-0.169	0.884	0.216
Phosphatidylcholine acyl-alkyl C44:4 [μmol/L]	0.55	0.45	0.55	0.12	0.47	0.33	0.49	0.11	-0.08	X	79	-1.099	0.287	0.059
Phosphatidylcholine acyl-alkyl C44:5 [μmol/L]	1.97	1.89	1.87	0.48	1.38	1.49	1.55	0.48	-0.59	X	67	-1.606	0.114	0.020
Phosphatidylcholine acyl-alkyl C44:6 [μmol/L]	1.37	1.04	1.29	0.28	1.06	0.89	1.15	0.33	-0.31	X	73	-1.352	0.186	0.029
**Sphingolipids**														
Hydroxysphingomyeline C14:1 [μmol/L]	6.08	6.53	6.74	1.91	7.52	6.8	7.34	1.97	1.44	X	76	-1.225	0.233	0.031
Hydroxysphingomyeline C16:1 [μmol/L]	3.44	3.34	3.7	0.95	4.52	4.09	4.31	1.20	1.08	X	65	-1.69	0.096	0.018
Hydroxysphingomyeline C22:1 [μmol/L]	7.25	8.01	7.88	2.07	8.72	6.72	8.65	1.86	1.47	X	72	-1.395	0.173	0.025
Hydroxysphingomyeline C22:2 [μmol/L]	7.13	7.45	7.47	1.82	7.93	7.24	8.04	1.98	0.80	X	76	-1.225	0.233	0.043
Hydroxysphingomyeline C24:1 [μmol/L]	0.65	0.82	0.66	0.18	0.68	0.47	0.73	0.14	0.03	X	75	-1.268	0.217	0.036
Sphingomyeline C16:0 [μmol/L]	121.95	78.9	123.69	18.26	137.69	96.65	131.77	27.79	15.74	X	74	-1.31	0.201	0.032
Sphingomyeline C16:1 [μmol/L]	16.45	11.13	16.5	2.81	17.26	13.97	17.71	4.41	0.81	X	83	-0.93	0.37	0.092
Sphingomyeline C18:0 [μmol/L]	20.96	20.59	22.12	4.87	25.21	24.38	25.25	7.21	4.25	X	61	-1.859	0.065	0.013
Sphingomyeline C18:1 [μmol/L]	8.65	9.37	8.82	1.97	9.92	11.39	10.36	3.5	1.27	X	70	-1.479	0.147	0.023
Sphingomyeline C20:2 [μmol/L]	0.37	0.38	0.36	0.10	0.35	0.36	0.38	0.1	-0.02	X	98	-0.296	0.787	0.203
Sphingomyeline C24:0 [μmol/L]	14.24	11.72	15.02	3.02	16.39	10.93	15.5	3.19	2.15	X	94	-0.465	0.663	0.165
Sphingomyeline C24:1 [μmol/L]	38.12	25.91	37.07	5.72	38.13	23.78	37.2	7.34	0.01	X	93	-0.507	0.633	0.155
Sphingomyeline C26:1[μmol/L]	0.2	0.39	0.21	0.1	0.19	0.33	0.22	0.09	-0.01	X	104	-0.042	0.983	0.225
**Sugars**														
Hexose [μmol/L]	4400.9	2320.2	4589.83	662.92	4394.96	2757.6	4635.0	868.07	-6.00		101	-0.169	0.884	0.218

^1^ BDI: Beck-Depression Inventory

^2^ HAMD-17: Hamilton Depression Scale-17

^3^ Difference of Median values: Median of patients with depression—Median of patients without depression

^4^ Benjamini Hochberg critical value for multiple comparisons: (i/m)xQ; i = the individual p-value’s rank, m = total number of tests, Q = false discovery rate: 0.25

^5^ Range is the difference between the largest and the smallest value

^●^ Analytes with levels between the lower limit of quantification (LOQ) and the limit of detection (LOD)

In addition to the metabolomics analysis we also measured alkaline phosphatase, gamma-glutamyltransferase, aspartate transaminase (ASAT), alanine transaminase (ALAT), cholinesterase and bilirubin with routine methods on a Roche COBAS C411 automated analyser (Roche Diagnostics, Mannheim, Germany). Thyroid-stimulating hormone, free thyroxine and free triiodothyronine concentrations have been analyzed by competitive immunoassay and direct chemiluminescence technology on an ADVIA Centaur XP automated analyser (Siemens Healthcare Diagnostics Inc., Tarrytown, NY, U.S.A.).

### Statistical analyses

Descriptive statistics are presented as mean ± standard deviation (SD), median, difference of medians and range. Group differences for categorical variables (gender, way of transmission, type of hepatitis C infection, type of interferon) were assessed using the Fisher’s exact test. Continuous variables (results of the depression questionnaires, laboratory values) were analysed by the Mann-Whitney-U test. Adjustment of p-values for multiple testing was performed where indicated using the Benjamini–Hochberg procedure, with a false discovery rate of 0.25 for screening experiments [18, 19]. All statistical analyses were performed with SPSS 22.0 for Windows (IBM-SPSS Statistics).

## Results

### Sociodemographic, clinical and treatment characteristics

The 31 participants of the study included 14 (45.2%) females and 17 (54.8%) males with a mean age of 43.5 (±14.4) years and 46.1 (±12.6) years respectively. Table 2 summarizes the sociodemographic, clinical and treatment characteristics such as the subtype of HCV infection, the presumed means of transmission and the type of interferon.

**Table 2:** Sociodemographic-, hepatitis C-, and treatment characteristics.

	All patients (n = 31)	Patients with Depression (n = 10)	Patients without Depression (n = 21)	p
**Sociodemographic characteristics**				
**Gender**				
Male	17 (54.8%)	6 (60.0%)	13 (61.9%)	p exact = 0.441 ^1^
Female	14 (45.2%)	4 (40.0%)	8 (38.1%)	
**Age**				
Mean (years)	44.9	47.05	43.9	Mann-Whitney-U Test: 88.5; U = -0.698, p exact = 0.492;
SD	±13.3	±14.7	±12.7	
Median	46.0	52.0	46.0	
Range ^2^	46.0	43.0	45.0	
**Hepatitis C infection characteristics**				
**Way of transmission**				
Unknown	13 (41.9%)	2 (20.0%)	11 (52.4%)	p exact = 0.271 ^1^
History of transfusion	13 (41.9%)	6 (60.0%)	7 (33.3%)	
Intravenous drug abuse	5 (16.1%)	2 (20.0%)	3 (14.3%)	
**Type of hepatitis C infection**				
HCV Type 1	8 (25.8%)	1 (10.0%)	7 (33.3%)	p exact = 0.204 ^1^
HCV Type 1a	7 (22.6%)	4 (40.0%)	3 (14.3%)	
HCV Type 1b	12 (38.7%)	3 (30.0%)	9 (42.9%)	
HCV Type 3	3 (9.7%)	1 (10.0%)	2 (9.5%)	
HCV Type 4	1 (3.2%)	1 (10.0%)	0 (0%)	
**Treatment characteristics**				
**Type of interferon**				
Peg Interferon α-2a	23 (74.2%)	8 (80.0%)	15 (71.4%)	p exact = 1.00 ^1^
Peg Interferon α-2b	8 (25.8%)	2 (20.0%)	6 (28.6%)	

SD = Standard deviation

^1^ Fisher’s exact test

^2^ Range is the difference between the largest and the smallest value

### IFN-α induced depression

Although the total scores of the depression scale HAMD-17 were elevated marginally in three patients before IFN-α therapy, they indicated only minor degrees of depressed mood in these patients before the start of treatment. One month after the initial IFN-α treatment the psychiatric assessments, based on a clinical psychiatric interview and supported by the HAMD-17 and BDI scales, identified 10 (32.3%) patients with treatment-related depression. These patients had a mean baseline HAMD-17 score of 6.1 (±5.3), which increased to 15.0 (±3.6) one month after the initial IFN-α treatment (Wilcoxon Test: -2.805, p< 0.005). Non-depressive patients had a baseline HAMD-17 score of 2.9 (±3.1), which remained at 3.9 (±2.3) one month after the initial IFN-α treatment (Wilcoxon Test: -3.135, p< 0.002).

Table 2 summarizes the sociodemographic, clinical and treatment characteristics of the depressive and non-depressive patients. Table 1 provides the HAMD-17 and BDI scores.

### Laboratory analyses

Metabolomic analysis identified the amino acid isoleucine as the only compound that differed significantly between depressive and non-depressive patients (Table 1). Depressive patients had a median isoleucine concentration of 51.35 (43.4–60.2) μmol/L that was significantly lower than in non-depressive subjects 62.1 (38.4–81.7) μmol/L (U = -2.958; p = 0.002). All other amino acids, acylcarnitines, biogenic amines, glycerophospholipids, sphingolipids, sugars, liver enzymes and thyroid levels did not differ between the two groups.

## Discussion

Metabolomic assessment of the biochemical signature of IFN-α treated HCV patients identified a decline of the branched chain amino acid isoleucine as a potential surrogate marker for immune-related depression. The absence of significant effects for any of the other molecules that were measured in parallel may highlight the particular role of isoleucine in the pathogenesis of immune-related depression.

The finding of decreased isoleucine levels in patients with immune-related depression is consistent with the results of a previous study by our group showing a significant reduction of the total sum of tryptophan-competing amino acids after the onset of IFN-α induced depression [5]. In addition, Capuron et al. [20] reported a significant decline of large neutral amino acids during the first four weeks of pegIFN-α therapy. Another comparison of somatically healthy depressive patients and mentally healthy controls showed significantly lower concentrations of isoleucine in depressed subjects [21]. Additional support for an involvement of isoleucine in depression comes from animal studies. Treatment of mice with paroxetine, a selective serotonin reuptake inhibitor (SSRI), increases the blood concentration of isoleucine, valine and leucine by 50–70% [22]. However, not all previous studies found lower isoleucine concentrations in depressed patients. Compared to 22 non-depressive controls, Woo et al. [23] reported comparable isoleucine concentrations in 68 depressed individuals before and six weeks after commencement of SSRI pharmacotherapy.

There are several pathways through which isoleucine could drive immune-related depression. Aquilani et al. [24] suggested that the oxidative degradation of BCAAs causes alterations of Krebs cycle intermediates, which may impact neurotransmitter synthesis. Lower concentrations of isoleucine may also dysregulate the mammalian target of rapamycin (mTOR) pathway facilitating the occurrence of immune-related depressive episodes. BCAAs, especially leucine, are well-known activators of mTOR [25]. Previous studies have shown that the mTOR pathway is activated in peripheral blood cells of depressed patients after acute sub-anaesthetic administration of ketamine, a NMDA receptor antagonist with well-known anti-depressive properties [26]. Diaz Granados et al. [27] reported that ketamine might even decrease suicidal ideation in treatment-resistant patients with major depression. In contrast, the inhibition of mTOR by rapamycin reverses the antidepressant effects of ketamine [28]. These findings suggest that an activation of mTOR improves depressive symptoms in patients with major depression. In view of these results we hypothesize that lower concentrations of isoleucine might be indicators of lower mTOR activation in depressive HCV patients, resulting in depressive symptomatology.

Lastly, isoleucine might also have an impact on the kynurenine pathway in patients with immune-related depression. A low isoleucine concentration might result in a greater kynurenine uptake from the blood stream into the astrocytes. The uptake of kynurenine by astrocytes occurs via large neutral amino acids transporters (LATs), which also transport BCAAs (e.g. isoleucine) and aromatic amino acids. Therefore it can be speculated that lower isoleucine concentrations result in less competition for LATs facilitating the uptake of kynurenine into the astrocytes and the production of kynurenic acid. Nanomolar increases of kynurenic acid might contribute to cognitive dysfunction, a common feature of depression [29, 30].

As described above, low levels of isoleucine might aggravate depression; however, it needs to be mentioned that high concentrations of isoleucine might have the opposite effect because the serotonin precursor tryptophan as well as isoleucine make use of the same transport system to cross the blood-brain barrier, and competition for the carrier protein might be the result [31]. The clinical impact of this competitive effect of isoleucine is controversially discussed [9], and further studies might address the best isoleucine balance.

### Limitations

In screening experiments with small sample sizes the risk of Type I errors might be increased.

There are also analytical aspects that have to be considered when interpreting the result of this project. For several analytes the sensitivity of the metabolomics method was insufficient to generate quantitative results. Therefore, we may have missed relevant differences for analytes that are present at low concentrations. Furthermore, the high cost of the Biocrates Absolute IDQ p180 kit made a structured assessment of its analytical performance not feasible. Data on imprecision, LoD and LoQ is only available from the pack insert provided by the manufacturer. Therefore it would be desirable to confirm our results with compound specific targeted methods that harbour an optimized analytical performance.

While previous studies have found great support for the inflammatory hypothesis of depression in depressive states with preceding immune activation, the impact of the inflammatory hypothesis of depression might be less important in somatically healthy depressive patients without such a previous immune activation [32].

## Conclusions

The activation of the Kynurenine-Quinolinic acid cascade is the well-established basis of the inflammatory hypothesis of depression. The results of this study now demonstrate that inflammatory processes might have further important consequences such as an isoleucine reduction. Thus, the results of this metabolomic pilot study identified isoleucine as a potential biomarker that is reduced in patients with immune-modulated major depression. This result should be confirmed in a prospective study that uses a targeted assay for BCAAs. Experimental studies should investigate if isoleucine plays a mechanistic role in the pathogenesis of major depression.

## Supporting information

S1 Dataset (XLS)

## Abbreviations

3-HK: 3-hydroxykynurenine
BCAA: branched-chain amino acid
BDI: Beck Depression Inventory
DAAs: directly acting antivirals
HAMD-17: Hamilton Rating Scale for Depression -17
HCV: hepatitis C virus
IDO: indoleamine 2,3-dioxygenase
IFN-α: interferon-α
LATs: large neutral amino acids transporters
mhGAP: Mental Health Gap Action Programme
NMDA: N-methyl-D-aspartate
pegIFN-α 2a: pegylated interferon alfa-2a
pegIFN-α 2b: pegylated interferon alfa-2a
PIRD: pharmacologically induced immune-related depression
SD: standard deviation
SSRI: selective serotonin reuptake inhibitor
TNF-α: tumor necrosis factor-α

## References

[1] World Health Organization. Depression. Fact sheet 369 (updated February 2017). http://www.who.int/mediacentre/factsheets/fs369/en/.

[2] American Psychiatric Association. Major Depressive Disorder in Diagnostic and Statistical Manual of Mental Disorders (5th ed.). Arlington, VA: American Psychiatric Association; 2013 pp 160–168.

[3] Rothenhäusler HB, Stepan A, Kreiner B, Baranyi A, Kapfhammer HP. Patterns of psychiatric consultation in an Austrian tertiary care center—results of a systematic analysis of 3,307 referrals over 2 years. Psychiatr Danub. 2008;20, 301–309. 18827755

[4] Ferrari F, Villa RF. The Neurobiology of Depression: an Integrated Overview from Biological Theories to Clinical Evidence. Mol Neurobiol. 2017;54, 4847–4865. 10.1007/s12035-016-0032-y 27510505 10.1007/s12035-016-0032-y

[5] Baranyi A, Meinitzer A, Stepan A, Putz-Bankuti C, Breitenecker RJ, Stauber R, et al A biopsychosocial model of interferon-alpha-induced depression in patients with chronic hepatitis C infection. Psychother Psychosom. 2013;82, 332–340. 10.1159/000348587 23942342 10.1159/000348587

[6] Baranyi A, Meinitzer A, Breitenecker RJ, Amouzadeh-Ghadikolai O, Stauber R, Rothenhäusler HB. Quinolinic Acid Responses during Interferon-α-Induced Depressive Symptomatology in Patients with Chronic Hepatitis C Infection—A Novel Aspect for Depression and Inflammatory Hypothesis. PloS One. 2015;10, e0137022 10.1371/journal.pone.0137022 26368809 10.1371/journal.pone.0137022 PMC4569409

[7] Raison CL, Borisov AS, Broadwell SD, Capuron L, Woolwine BJ, Jacobson IM, et al Depression during pegylated interferon-alpha plus ribavirin therapy: Prevalence and prediction. J Clin Psychiatry. 2005;66, 41–48. 15669887 10.4088/jcp.v66n0106 PMC1615913

[8] Neumeister A, Yuan P, Young TA, Bonne O, Luckenbaugh DA, Charney DS, et al Effects of tryptophan depletion on serum levels of brain-derived neurotrophic factor in unmedicated patients with remitted depression and healthy subjects. Am J Psychiatry. 2005;162, 805–807. 10.1176/appi.ajp.162.4.805 15800160 10.1176/appi.ajp.162.4.805

[9] Wichers MC, Koeck GH, Robaeys G, Verkerk R, Scharpé S, Maes M. IDO and interferon-alpha-induced depressive symptoms: A shift in hypothesis from tryptophan depletion to neurotoxicity. Mol Psychiatry. 2005;10, 538–544. 10.1038/sj.mp.4001600 15494706 10.1038/sj.mp.4001600

[10] Bonaccorso S, Marino V, Biondi M, Grimaldi F, Ippoliti F, Maes M. Depression induced by treatment with interferon-alpha in patients affected by hepatitis C virus. J Affect Disord. 2002;72, 237–241. 12450640 10.1016/s0165-0327(02)00264-1

[11] Bonaccorso S, Marino V, Puzella A, Pasquini M, Biondi M, Artini M, et al Increased depressive ratings in patients with hepatitis C receiving interferon-α based immunotherapy are related to interferon-α induced changes in the serotonergic system. J Clin Psychopharmacol. 2002;22, 86–90. 11799348 10.1097/00004714-200202000-00014

[12] World Health Organization. Global report on access to hepatitis C treatment—Focus on overcoming barriers. http://apps.who.int/iris/bitstream/10665/250625/1/WHO-HIV-2016.20-eng.pdf?ua=1 (2016).

[13] Baranyi A, Meinitzer A, Putz-Bankuti C, Stauber R, Kapfhammer HP, Rothenhäusler HB. Asymmetric dimethylarginine responses during interferon-α-induced depression in patients with chronic hepatitis C infection. Psychosom Med. 2014;76, 197–207. 10.1097/PSY.0000000000000042 24608038 10.1097/PSY.0000000000000042

[14] Baranyi A, Amouzadeh-Ghadikolai O, Lewinski DV, Breitenecker RJ, Stojakovic T, März W, et al Beta-trace Protein as a new non-invasive immunological Marker for Quinolinic Acid-induced impaired Blood-Brain Barrier Integrity. Sci Rep. 2017;7, 43642 10.1038/srep43642 28276430 10.1038/srep43642 PMC5343478

[15] Hamilton M. Development of a rating scale for primary depressive illness. Br J Soc Clin Psychol. 1967;6, 78–296. 10.1111/j.2044-8260.1967.tb00530.x 6080235

[16] Beck AT, Ward CH, Mendelson M, Mock J, Erbaugh J. An inventory for measuring depression. Arch Gen Psychiatry. 1961;4, 561–571. 13688369 10.1001/archpsyc.1961.01710120031004

[17] Biocrates Life Sciences. Absolute IDQ p180kit. Analytical specifications p180 (AS-p180-2 for research use) Innsbruck: Biocrates Life Sciences; 2010.

[18] Hochberg Y, Benjamini Y. More powerful procedures for multiple significance testing. Stat Med. 1990;9, 811–818. 2218183 10.1002/sim.4780090710

[19] McDonald JH. Handbook of Biological Statistics (3rd ed.). Baltimore, Maryland, U.S.A.: Sparky House Publishing; 2014.

[20] Capuron L, Ravaud A, Neveu PJ, Miller AH, Maes M, Dantzer R Association between decreased serum tryptophan concentrations and depressive symptoms in cancer patients undergoing cytokine therapy. Mol Psychiatry. 2002;7, 468–473. 10.1038/sj.mp.4000995 12082564 10.1038/sj.mp.4000995

[21] Baranyi A, Amouzadeh-Ghadikolai O, von Lewinski D, Rothenhäusler HB, Theokas S, Robier C, et al Branched-Chain Amino Acids as New Biomarkers of Major Depression—A Novel Neurobiology of Mood Disorder. Plos One. 2016;11(8), e0160542 10.1371/journal.pone.0160542 27490818 10.1371/journal.pone.0160542 PMC4973973

[22] Webhofer C Gormanns P, Tolstikov V, Zieglgänsberger W, Sillaber I, Holsboer F, et al Metabolite profiling of antidepressant drug action reveals novel drug targets beyond monoamine elevation. Transl Psychiatry. 2011;1: e58 10.1038/tp.2011.56 . 22832350 10.1038/tp.2011.56 PMC3309495

[23] Woo HI, Chun MR, Yang JS, Lim SW, Kim MJ, Kim SW, et al Plasma amino acid profiling in major depressive disorder treated with selective serotonin reuptake inhibitors. CNS Neurosci Ther. 2015;21, 417–424. 10.1111/cns.12372 25611566 10.1111/cns.12372 PMC6495833

[24] Aquilani R, Boselli M, Boschi F, Viglio S, Iadarola P, Dossena M, et al Branched-chain amino acids may improve recovery from a vegetative or minimally conscious state in patients with traumatic brain injury: a pilot study. Arch Phys Med Rehabil. 2008; 89, 1642–1647. 10.1016/j.apmr.2008.02.023 18760149 10.1016/j.apmr.2008.02.023

[25] Dyachok J, Earnest S, Iturraran EN, Cobb MH, Ross EM. Amino Acids Regulate mTORC1 by an Obligate Two-step Mechanism. J Biol Chem. 2016;291, 22414–22426. 10.1074/jbc.M116.732511 27587390 10.1074/jbc.M116.732511 PMC5077182

[26] Yang C, Zhou ZQ, Gao ZQ, Shi JY, Yang JJ. Acute increases in plasma mammalian target of rapamycin, glycogen synthase kinase-3b, and eukaryotic elongation factor 2 phosphorylation after ketamine treatment in three depressed patients. Biol Psychiatry. 2013; 73,35–36. 10.1016/j.biopsych.2012.07.022 22884969

[27] Diaz Granados N, Ibrahim LA, Brutsche NE, Ameli R, Henter ID, Luckenbaugh DA, et al Rapid resolution of suicidal ideation after a single infusion of an N-methyl-D-aspartate antagonist in patients with treatment-resistant major depressive disorder. J Clin Psychiatry. 2010; 71,1605–1611. 10.4088/JCP.09m05327blu 20673547 10.4088/JCP.09m05327blu PMC3012738

[28] Yu JJ, Zhang Y, Wang Y, Wen ZY, Liu XH. Qin J, et al Inhibition of calcineurin in the prefrontal cortex induced depressive-like behavior through mTOR signaling pathway. Psychopharmacology. 2013;225, 361–372. 10.1007/s00213-012-2823-9 22875481 10.1007/s00213-012-2823-9

[29] Sekine A, Okamoto M, Kanatani Y, Sano M, Shibata K, Fukuwatari T. Amino acids inhibit kynurenic acid formation via suppression of kynurenine uptake or kynurenic acid synthesis in rat brain in vitro. Springerplus. 2015;4, 48 10.1186/s40064-015-0826-9 25674503 10.1186/s40064-015-0826-9 PMC4318830

[30] Chess AC, Simoni MK, Alling TE, Bucci DJ. 2007 Elevations of endogenous kynurenic acid produce spatial working memory deficits. Schizophr Bull. 2007;33, 797–804. 10.1093/schbul/sbl033 16920787 10.1093/schbul/sbl033 PMC2526148

[31] Lucca A, Lucini V, Catalano M, Alfano M, Smeraldi E. Plasma tryptophan to large neutral amino acids ratio and therapeutic response to a selective serotonin uptake inhibitor. Neuropsychobiology. 1994;29, 108–111. 10.1159/000119071 8022529 10.1159/000119071

[32] Baranyi A., Amouzadeh-Ghadikolai O, von Lewinski D, Breitenecker RJ, Rothenhäusler HB, Robier C, et al Revisiting the tryptophan-serotonin deficiency and the inflammatory hypotheses of major depression in a biopsychosocial approach. PeerJ. 2017;5, e3968 10.7717/peerj.3968 29109914 10.7717/peerj.3968 PMC5671663

